# Absolute structure of the chiral pyrrolidine derivative (2*S*)-methyl (*Z*)-5-(2-*tert*-but­oxy-1-cyano-2-oxo­ethyl­idene)pyrrolidine-2-carboxyl­ate, a com­pound with low resonant scattering

**DOI:** 10.1107/S2053229619012324

**Published:** 2019-10-04

**Authors:** Ai Wang, Ulli Englert

**Affiliations:** a Key Laboratory of Materials for Energy Conversion and Storage, Institute of Molecular Science, Shanxi University, Taiyuan, Shanxi 030006, People’s Republic of China; b Institute of Inorganic Chemistry, RWTH Aachen University, Landoltweg 1, 52074 Aachen, Germany

**Keywords:** pyrrolidine derivative, absolute structure, resonant scattering, circular dichroism, crystal structure

## Abstract

The enanti­opure monopyrrolidine derivative (2*S*)-methyl (*Z*)-5-(2-*tert*-but­oxy-1-cyano-2-oxo­ethyl­idene)pyrrolidine-2-carboxyl­ate, C_13_H_18_N_2_O_4_, (**1**), represents a potential ligand and an attractive inter­mediate for the synthesis of chiral metal com­plexes. At the mol­ecular level, the com­pound features an intra­molecular N—H⋯O hydrogen bond; neighbouring mol­ecules inter­act *via* N—H⋯N contacts to form chains along [100]. Due to its elemental com­position, resonant scattering of the target com­pound is entirely insignificant for diffraction experiments with Mo *K*α and small even for Cu *K*α radiation. A preliminary study with the harder radiation type confirmed the chiral space group and the suitability of the single crystal chosen; as expected, the results concerning the absolute structure remained com­pletely inconclusive. A second data collection with the longer wavelength gave satisfactory quality indicators for the correct handedness of the mol­ecule, albeit with high standard uncertainties. The absolute configuration has been assessed independently: CD spectra for both enanti­omers of the target mol­ecule were calculated and the spectrum for the *S*-configured stereoisomer was in agreement with the experiment. The Cotton effect of (**1**) may be ascribed to π–π* transitions from HOMO to LUMO and from HOMO to LUMO+1. As both independent techniques agree with respect to the handedness of the target mol­ecule, the absolute structure may be assigned with a high degree of confidence.

## Abstract 2

The light-atom com­pound (2*S*)-methyl (*Z*)-5-(2-*tert*-but­oxy-1-cyano-2-oxo­ethyl­idene)pyrrolidine-2-carboxyl­ate is an enanti­opure coordination partner for cations. Despite its only minor resonant scattering, the absolute structure was determined by a combination of diffraction, CD spectroscopy and theoretical calculations.

## Introduction   

Pyrrolidine derivatives have found applications as potential ligands, as organic inter­mediates and in medicinal chemistry. They can inhibit the activity of over-expressed protein tyrosine phosphatases (PTPs) of cancer cells and may be employed as anti­cancer drugs (IC_50_ value is 3.65 ± 0.08 µ*M*) (Chen *et al.*, 2017 ▸). By forming imine or enamine inter­mediates with aldehydes and ketones, chiral monopyrrolidine derivatives have been widely used in asymmetric catalysis, and alkyl­ation and acyl­ation reactions of aldehydes and ketones have been achieved (Jensen *et al.*, 2012 ▸). We report here the absolute configuration of the chiral pyrrolidine derivative (2*S*)-methyl (*Z*)-5-(2-*tert*-but­oxy-1-cyano-2-oxo­ethyl­idene)pyrrolidine-2-carboxyl­ate, (**1**) (Scheme 1). The com­pound has been synthesized and spectroscopically characterized by Pfaltz and co-workers (Pfaltz *et al.*, 1977 ▸; Fritschi *et al.*, 1988 ▸; Pfaltz, 1993 ▸); retention of the configuration at C1 may be assumed. No studies in medicinal chemistry have been conducted on (**1**), but a closely related com­pound was investigated, *i.e.* methyl 5-[1-cyano-2-oxo-2-(2,3,4-tri­meth­oxy­phen­yl)ethyl­idene]pro­lin­ate was screened by the National Cancer Institution, USA, against 60 human tumour cell lines and showed moderate cell-growth inhibition at 10 µ*M* concentration for renal cancer and leukemia 
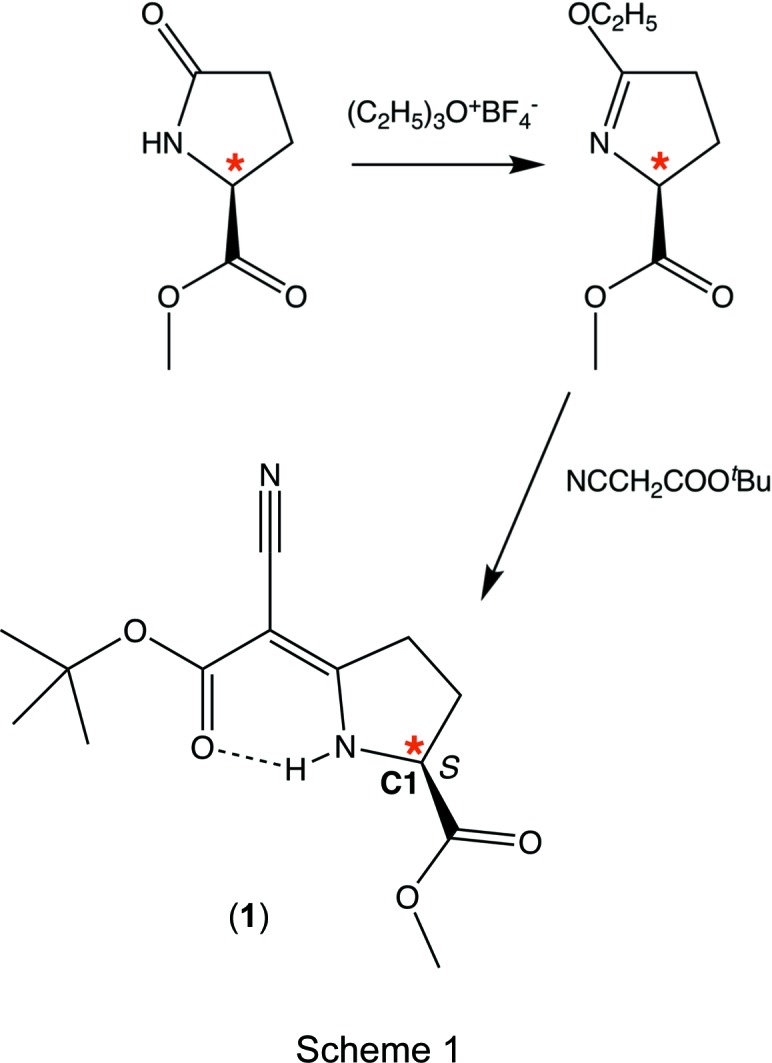
(Ghinet *et al.*, 2012 ▸). To the best of our knowledge, the structure of (**1**) has never been investigated and its absolute configuration has not been confirmed. Our assignment relies on a combination of diffraction experiments, experimental circular dichroism (CD) spectroscopy and theoretical calculations of these spectra. We will show that diffraction results, albeit with only a modest contribution of resonant scattering, and CD spectroscopy agree in their assignment of the absolute structure, whereas a diffraction experiment without relevant anomalous dispersion remains inconclusive.

## Experimental   

### Synthesis and crystallization   

All reagents were commercially available and were used without further purification. The powder diffraction experiment was recorded at the Institute of Inorganic Chemistry, RWTH Aachen University, using a Stoe imaging-plate detector (IP–PSD). The diffractogram was recorded on a flat sample at ambient temperature in transmission mode using Cu *K*α_1_ radiation. The title com­pound was synthesized following the procedure of Pfaltz (Pfaltz *et al.*, 1977 ▸; Fritschi *et al.*, 1988 ▸; Pfaltz, 1993 ▸). The reaction combines *S*-configured pyroglutamic acid methyl ester and *tert*-butyl 2-cyano­acetate; retention of the configuration at the chiral centre (*) was expected [see Scheme 1 for a summary of the synthesis for (**1**) according to Pfaltz *et al.* (1977 ▸)] and is confirmed by the results reported in this work.

**Figure 1:**
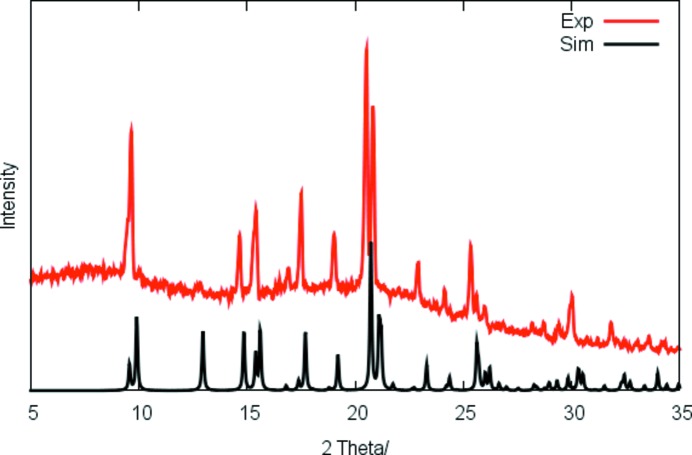
Powder X-ray diffraction pattern of (**1**).

Crystals were grown by slow partial evaporation of a methanol solvent at ambient temperature over a period of one week. CHN microanalysis was carried out at the Institute of Organic Chemistry, RWTH Aachen University, using a HERAEUS CHNO-Rapid. Analysis calculated (%) for C_13_H_18_N_2_O_4_: C 58.74, H 6.81, N 10.52; found: C 58.62, H 6.53, N 10.72. The powder X-ray diffraction (PXRD) pattern (see Fig. 1 ▸) confirms that (**1**) is obtained as an essentially phase-pure product; the shift of the calculated lines of two larger angles can be attributed to the different data-collection tem­peratures for the single-crystal and powder analyses. The IR spectrum shows an absorption associated with the triple bond in the nitrile group at ν(C≡N) = 2205 cm^−1^, in good agreement with the reported frequency of 2207 cm^−1^, and the ^1^H NMR spectrum matches that available in the literature (Fritschi *et al.*, 1988 ▸).

### Refinement   

Crystal data, data collection parameters and refinement results for both single-crystal X-ray diffraction experiments with Mo *K*α (**1mo**) and Cu *K*α (**1cu**) radiation are summarized in Table 1 ▸. H atoms attached to C atoms were introduced in calculated positions and treated as riding, with *U*_iso_(H) = 1.5*U*_eq_(C) for CH_3_ groups and 1.2*U*_eq_(C) otherwise. For the H atom attached to an N atom, the positional coordinates and an isotropic displacement parameter were refined freely. For the diffraction experiment (**1mo**), resonant scattering is insignificant; no information can de deduced from the refined enanti­opol parameter and its very high standard uncertainty. For a better com­parison with the results of (**1cu**) on the same single crystal, the same absolute structure model was chosen in both cases.

### CD spectroscopy   

The experimental electronic circular dichroism (CD) spectrum of (**1**) was recorded in methanol on a Chirascan circular dichroism chiroptical spectrometer at the Institutes of Biomedical Sciences of Shanxi University; it shows a positive Cotton effect at 278.40 nm and a negative Cotton effect at 245.60 nm.

## Results and discussion   

### Mol­ecular structure   

The chiral com­pound (**1**) was obtained as an essentially monophasic crystalline product. In view of its elemental com­position, the determination of the absolute structure was expected to be challenging. With respect to resonant scattering, we calculated values of 6 and 33 for *Friedif* (Flack & Shmeuli, 2007 ▸) for diffraction experiments with Mo and Cu *K*α radiation, respectively. Even the higher second value is dangerously low if the diffraction experiments are hampered by additional com­plications, such as disorder or twinning. An initial data collection with our standard set-up (**1mo**) was performed to ensure sufficient quality for the selected crystal and to confirm the chiral space group: even for high enan­tio­meric excesses, a small amount of racemic solid might precipitate (Böhme & Fels, 2013 ▸).

**Figure 2:**
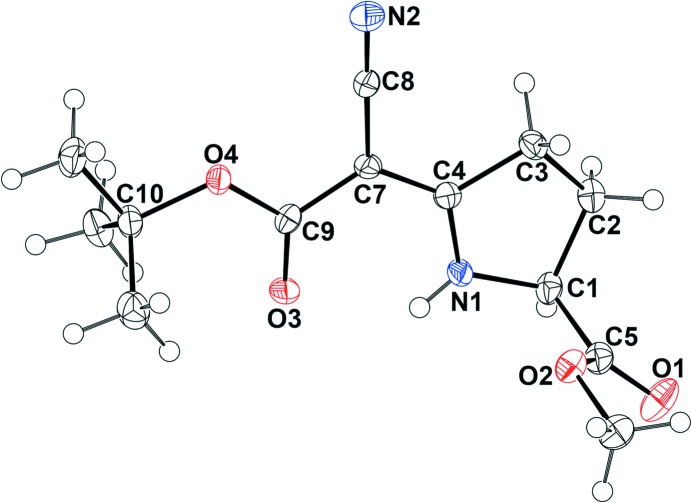
The asymmetric unit of (**1**) based on data set (**1cu**), with displacement ellipsoids enclosing 50% of electron density.

The second data set collected with Cu *K*α radiation resulted in slightly smaller standard uncertainties; all numerical values reported below therefore refer to (**1cu**) (see §3.3). As expected, the enanti­opure com­pound (**1**) crystallized in a chiral space group. The asymmetric unit consists of a single mol­ecule in the space group *P*2_1_2_1_2_1_; Fig. 2 ▸ shows a displacement ellipsoid plot.

**Figure 3:**
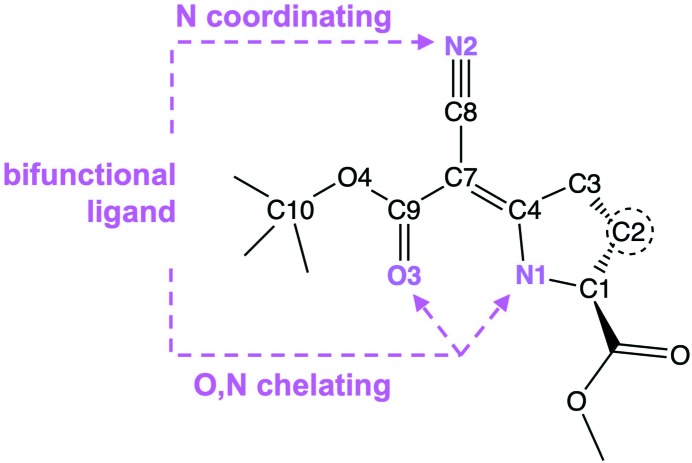
The planar core of (**1**).

Atoms N1, N2, O3, O4, C3, C4 and C7–C10 define an almost planar core of the mol­ecule shown in Fig. 3 ▸. The maximum deviation from that least-squares plane is 0.045 (2) Å for atom C3. Within the core plane, the pyrrolidine N—H group acts as a hydrogen-bond donor towards carbonyl atom O3; the hydrogen-bond geometry is summarized in Table 2 ▸.

The pyrrolidine ring is nonplanar and its C2 atom is significantly displaced from the above-defined plane by 0.364 (2) Å.

In contrast to the carboxyl­ate group (C9/O3/O4), the C5/O1/O2 group is not coplanar with the core of the mol­ecule but subtends an angle of 86.1 (2)° with the least-squares plane defined by atoms N1, N2, O3, O4, C3, C4 and C7–C10 (Fig. 2 ▸). Table 3 ▸ contains selected torsion angles.

The overall conformation of the mol­ecule suggests its use as a ditopic ligand, similar to substituted acetyl­acetones (Kremer & Englert, 2018 ▸). The potential coordination sites have been indicated in Fig. 3 ▸.

### Inter­molecular contacts   

**Figure 4:**
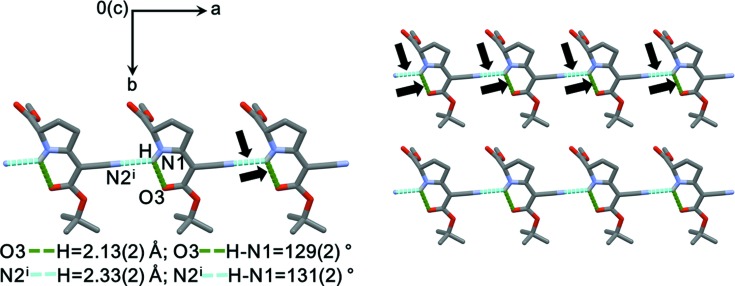
Intra- and inter­molecular hydrogen bonds in the crystal of (**1**). H atoms not involved in hydrogen bonds have been omitted for clarity.

The H atom of the pyrrolidine N—H group represents the only potential donor for classical hydrogen bonds. In addition to the intra­molecular N—H⋯O contact described above, it is involved in an inter­molecular N—H⋯N hydrogen bond to the nitrile group of a neighbouring mol­ecule. The resulting chain runs along [100] (Fig. 4 ▸). The closest contacts perpendicular to this chain are due to nonclassical C—H⋯O inter­actions. Numerical values and symmetry operators for the short contacts have been com­piled in Table 2 ▸.

### Absolute structure   

#### Resonant scattering   

Our first intensity data collection, *i.e.* the (**1mo**) data, had provided a consistent structure model without disorder and confirmed the quality of the chosen sample. As expected, however, the commonly applied methods for assigning the absolute structure gave inconclusive results for (**1mo**) with its negligible resonant scattering. The Flack (1983 ▸, 2003 ▸), Parsons (Parsons *et al.*, 2013 ▸) and Hooft (Hooft *et al.*, 2010 ▸) parameters refined to values of *ca* 1, with standard uncertainties equally large; no conclusions could be drawn from these numbers. Therefore, a second diffraction experiment with Cu *K*α radiation, *i.e.* the (**1cu**) data, was performed on the same single crystal. Fractional coordinates and derived geometry parameters agreed with the results of (**1mo**) within error, but resonant scattering was more pronounced and led to information about the absolute structure, *i.e.* the Flack (1983 ▸) parameter refined to −0.04 (12); very similar values and standard uncertainties were obtained for Parsons’ quotient method [−0.01 (13), Parsons *et al.*, 2013 ▸] and Hooft’s Bayesian procedure [0.01 (10), Hooft *et al.*, 2010 ▸].

#### CD spectra   

**Figure 5:**
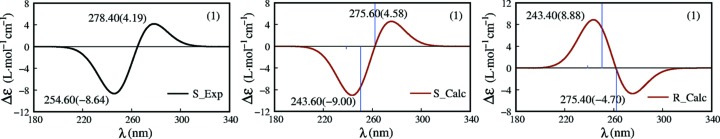
Experimental (left) and calculated CD spectra for (**1**) in methanol. The spectrum in the centre corresponds to the (correct) *S* enanti­omer and that on the right to the alternative *R* enanti­omer.

An independent assessment of the absolute structure of (**1**) relies on a com­parison of the experimentally observed and theoretically calculated electronic circular dichroism (ECD) spectra; they are shown in Fig. 5 ▸.

The calculations were based initially on the mol­ecular geometry obtained from (**1cu**). Ground-state geometry optimization and subsequent frequency calculations were performed *via* the density functional theory (DFT) method as implemented in *GAUSSIAN09* (Frisch *et al.*, 2009 ▸) using the B3LYP hybrid functional (Becke, 1993 ▸) and the 6-311++G(2d,p) basis set. The excitation energies, oscillator and rotational strengths of the excited singlet states for the optimized geometry were calculated according to the time-dependent DFT (TDDFT) method with the same functional and basis set. The effects of the solvent (methanol) were included using the polarizable continuum model (PCM) (Tomasi *et al.*, 2005 ▸) in the integral equation formalism (IEF). With the PCM, a ground-state energy of −916.95 a.u. for (**1**) was obtained.

#### DFT energy levels and Kohn–Sham orbitals   

**Figure 6:**
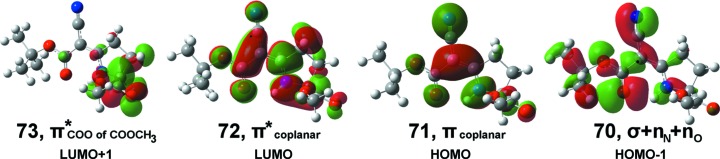
Selected Kohn–Sham orbitals for (**1**).

The DFT energy levels show a HOMU–LUMO gap of 5.25 eV. A detailed analysis of the Kohn–Sham orbitals has been graphically summarized in Fig. 6 ▸. The two lowest unoccupied orbitals are dominated by a π* region in the planar core and in the carboxyl­ate group of the methyl ester (C5/O1/O2). The absolute value of the energy difference between these LUMO and LUMO+1 orbitals is 0.72 eV. Both of them may well act as electron-acceptor orbitals when electrons from the HOMO and HOMO-1 orbitals are excited. The HOMO is dominated by the π region of the planar core of (**1**). The HOMO-1 essentially corresponds to a combination of σ + *n*_N_ + *n*_O_ orbitals; the energy difference of the HOMO and HOMO-1 amounts to 1.28 eV.

#### Rotational strengths and transition assignments   

The contribution of different transition probabilities to the chiroptical properties of (**1**) were analyzed. The calculated excitation energies and oscillator and rotational strengths (in velocity form), as well as the transition assignments, have been com­piled in Table 4 ▸. Results for the three excitations of the lowest energy conformer are given; they cover the spectral range 180 < λ < 350 nm.

Using the excitation energies and rotational strengths calculated by TDDFT, theoretical CD spectra for both stereoisomers of (**1**) were generated as the sum of Gaussians, centred at the calculated wavelengths λ_calc_ with integral intensities proportional to the rotational strengths *R* of the corresponding transitions. The half bandwidths Γ at the Δ∊_max_/e of Gaussians were assumed as Γ = *k*λ_calc_^3/2^ (Brown *et al.*, 1971 ▸) with *k* = 0.00385 to best reproduce the experiment. The experimental spectrum and calculated spectra for both enanti­omers have been com­piled in Fig. 5 ▸. Ideally, experimental CD spectra of opposite enanti­omers are mirror images of each other (Flack & Bernardinelli, 2003 ▸).

It is obvious that the CD curve calculated for *S*-configured (**1**) is in excellent agreement with the observed curve, with only a small blue shift in the calculated maximum. The agreement confirms that our spectroscopic inter­pretation of the DFT results is correct.

The observed CD curve consists of two absorption bands, *i.e.* a positive band around 278 nm arising from the first π–π* transition in which electrons are transferred from the HOMO to the LUMO (77%) and from the HOMO to the LUMO+1 (19%), and a negative band around 243 nm, which can also be ascribed to the second π–π* transition and a minor contribution of a σ–π* transition. The main contribution to this significant negative π–π* transition, however, is associated with the transition from HOMO to LUMO+1 (77%) and from HOMO to LUMO (20%). The σ–π* transition can be assigned to electronic excitation from HOMO-1 to LUMO. Thus, the optical properties of chiral com­pound (**1**) are mainly dominated by a combination of π–π* transitions from HOMO to LUMO and HOMO to LUMO+1.

## Conclusion and outlook   

The absolute structure of (**1**) could reliably be assigned as *S*, despite the limited contribution of resonant scattering; a low-temperature diffraction experiment with Cu *K*α radiation resulted in consistent values for the commonly applied enanti­opol parameters. Their final standard uncertainties are still rather high, but our assignment is in agreement with the expected retention at the stereocentre of the starting material and could be further corroborated by the match between experimentally observed and theoretically calculated CD spectra. The associated Cotton effect was well reproduced by our TDDFT calculations, thus confirming that our methodology was suitable. We hope to use enanti­opure (**1**) in future experiments as a ditopic ligand with the additional possibility to transfer central chirality from the ligand to its coordination com­plexes (Wang *et al.*, 2015 ▸).

## Supplementary Material

Crystal structure: contains datablock(s) 1mo, 1cu, global. DOI: 10.1107/S2053229619012324/yf3192sup1.cif

Structure factors: contains datablock(s) 1mo. DOI: 10.1107/S2053229619012324/yf31921mosup2.hkl

Structure factors: contains datablock(s) 1cu. DOI: 10.1107/S2053229619012324/yf31921cusup3.hkl

CCDC references: 1951413, 1951412

## Figures and Tables

**Table 1:** Experimental details For both determinations: C_13_H_18_N_2_O_4_, *M*_r_ = 266.29, orthorhombic, *P*2_1_2_1_2_1_, *Z* = 4. Experiments were carried out at 100 K using a D8 goniometer with an APEX CCD area detector. Absorption was corrected for by multi-scan methods (*SADABS*; Bruker, 2008 ▸). H atoms were treated by a mixture of independent and constrained refinement.

	(**1mo**)	(**1cu**)
Crystal data		
*a*, *b*, *c* (Å)	7.347 (4), 10.197 (6), 18.477 (10)	7.3731 (3), 10.1909 (4), 18.4972 (7)
*V* (Å^3^)	1384.1 (13)	1389.85 (9)
Radiation type	Mo *K*α	Cu *K*α
μ (mm^−1^)	0.10	0.79
Crystal size (mm)	0.35 × 0.29 × 0.28	0.35 × 0.29 × 0.28

Data collection		
*T*_min_, *T*_max_	0.473, 0.745	0.579, 0.753
No. of measured, independent and observed [*I* > 2σ(*I*)] reflections	10626, 2290, 2051	17945, 2408, 2266
*R*_int_	0.091	0.083
(sin θ/λ)_max_ (Å^−1^)	0.583	0.597

Refinement		
*R*[*F* ^2^ > 2σ(*F* ^2^)], *wR*(*F* ^2^), *S*	0.051, 0.124, 1.08	0.032, 0.077, 1.10
No. of reflections	2290	2408
No. of parameters	180	181
Δρ_max_, Δρ_min_ (e Å^−3^)	0.21, −0.18	0.17, −0.15
Absolute structure	Flack *x* determined using 710 quotients [(*I* ^+^) − (*I* ^−^)]/[(*I* ^+^) + (*I* ^−^)] (Parsons *et al.*, 2013 ▸)	Flack *x* determined using 879 quotients [(*I* ^+^) − (*I* ^−^)]/[(*I* ^+^) + (*I* ^−^)] (Parsons *et al.*, 2013 ▸)
Absolute structure parameter	1.1 (10)	−0.04 (12)

Computer programs: *SMART* (Bruker, 2001 ▸), *SAINT-Plus* (Bruker, 2009 ▸), *SHELXS2013* (Sheldrick, 2008 ▸), *SHELXL2017* (Sheldrick, 2015 ▸) and *PLATON* (Spek, 2009 ▸).

**Table 2:** Hydrogen-bond geometry (Å, °) for (**1cu**)

*D*—H⋯*A*	*D*—H	H⋯*A*	*D*⋯*A*	*D*—H⋯*A*
N1—H1N⋯O3	0.81 (3)	2.13 (2)	2.714 (2)	129 (2)
N1—H1N⋯N2^i^	0.81 (3)	2.33 (2)	2.924 (2)	131 (2)
C11—H11*B*⋯O2^ii^	0.98	2.60	3.565 (3)	169

Symmetry codes: (i) 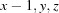; (ii) 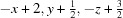.

**Table 3:** Selected torsion angles (°) for (**1cu**)

C4—N1—C1—C5	109.61 (19)	N1—C1—C5—O2	−26.3 (2)
C4—N1—C1—C2	−12.9 (2)	C2—C1—C5—O2	90.1 (2)
C1—N1—C4—C7	−179.84 (18)	C3—C4—C7—C8	−2.4 (3)
C1—N1—C4—C3	−0.6 (2)	N1—C4—C7—C9	−2.1 (3)
C2—C3—C4—N1	14.0 (2)	C10—O4—C9—O3	−2.1 (3)
C6—O2—C5—O1	−2.2 (3)	C4—C7—C9—O3	−1.4 (3)
C6—O2—C5—C1	179.93 (16)	C8—C7—C9—O4	0.4 (3)
N1—C1—C5—O1	155.73 (19)	C9—O4—C10—C13	61.8 (2)

**Table 4:** Excitation wavelengths (λ, nm), oscillator (*f*) and rotational (*R*, DBM) strengths and transition assignments from occupied (Occ) to virtual (Virt) orbitals

λ	*f*	*R*	Occ–Virt	Assignments
262	0.2670	0.4600	HOMO→LUMO	π(coplanar)→π*(coplanar) (77%)
			HOMO→LUMO+1	π(coplanar)→π*(COO in COOCH_3_) (19%)
250	0.1642	−0.6047	HOMO→LUMO+1	π(coplanar)→π*(COO in COOCH_3_) (77%)
			HOMO→LUMO	π(coplanar)→π*(coplanar) (20%)
239	0.0028	−0.0173	HOMO-1→LUMO	σ+*n*_N_+*n*_O_→π*(coplanar) (89%)
